# Influence of dementia literacy and caregiving appraisal on the psychological wellbeing of informal caregivers of people with dementia: A cross-sectional study

**DOI:** 10.3389/fmed.2022.971481

**Published:** 2022-09-14

**Authors:** Shanshan Wang, Qiuju Shan, Daphne Sze Ki Cheung, Xinyi Xu, Isaac Sze Him Leung, Angela Yee Man Leung

**Affiliations:** ^1^School of Nursing, The Hong Kong Polytechnic University, Kowloon, Hong Kong SAR, China; ^2^School of Nursing and Health, Zhengzhou University, Henan, China; ^3^WHO Collaborating Center for Community Health Services, School of Nursing, The Hong Kong Polytechnic University, Hong Kong, Hong Kong SAR, China; ^4^The Second Affiliated Hospital of Zhengzhou University, Henan, China; ^5^School of Nursing, Hebei Medical University, Hebei, China; ^6^Postdoctoral Research Station in Basic Medicine, Hebei Medical University, Hebei, China; ^7^Department of Statistics, The Chinese University of Hong Kong, Hong Kong, Hong Kong SAR, China

**Keywords:** dementia, literacy, caregiver, wellbeing, caregiving appraisal

## Abstract

**Background:**

Dementia informal caregiving is a global phenomenon. It is well documented that caregivers' psychological health is often affected by busy caregiving schedules. Lawton's two-factor model indicates that caregivers' psychological wellbeing is influenced by caregiving appraisal and other caregiver factors. Our review also identified the significance of dementia literacy, which was an essential caregiver factor. However, it is imperative for a clear understanding of the mechanism of how psychological wellbeing is influenced by them.

**Objectives:**

To explore the association among dementia literacy, caregiving appraisal, and psychological wellbeing and the influencing mechanisms between them.

**Methods:**

Two hundred and twenty-three informal caregivers of people with dementia were involved in this cross-sectional study. Dementia literacy was measured by the Alzheimer's Disease Knowledge Scale and Dementia Attitude Scale. Caregiving appraisal and psychological wellbeing were measured with the Caregiving Appraisal Scale and Ryff's Psychological wellbeing Scale, respectively. Descriptive statistics were used to describe the characteristics of participants and key outcome variables. Pearson's correlation analysis was used to analyze the correlation among the variables. Structural equation modeling was used to examine the hypothetical mediating role of caregiving appraisal in the relationship between dementia literacy (i.e., knowledge of dementia and attitude toward dementia) and caregivers' psychological wellbeing.

**Results:**

The hypotheses were partially confirmed. Attitude toward dementia was significantly associated with caregiving appraisal (*r* = 0.312, *p* < 0.01) and psychological wellbeing (*r* = 0.311, *p* < 0.01). However, knowledge of dementia was only significantly associated with psychological wellbeing (*r* = 0.136, *p* < 0.05). The structural equation modeling fitted well (*p* = 0.078, CFI = 0.987, RMSEA = 0.038). In the fitted model, caregiving appraisal partially mediated the association between attitude toward dementia and psychological wellbeing. In contrast, attitude toward dementia and caregiving appraisal fully mediated the association between knowledge of dementia and psychological wellbeing.

**Conclusion:**

Efforts can be exerted to improve dementia caregivers' caregiving appraisal and attitude toward dementia to improve their psychological wellbeing.

## Introduction

With the aging of society, dementia informal caregiving has become a global phenomenon. Most people with dementia are cared for by informal caregivers in the community (1–3). Informal caregivers are the individuals who voluntarily care for a relative or friend with illness or any condition that requires particular attention (4). Due to the long-term progression of the disease, informal caregivers need to provide consistent care to manage the complex behavioral and psychological syndromes of dementia and support their activities of daily living. Caregiving can affect caregivers' physical and psychosocial health. In recent years, much attention has been paid to the negative consequences of caregiving, such as the highly prevalent mental health problems of stress, anxiety, and depression (5), as well as higher morbidity and mortality (6). As caregiving is a complex process involving both positive and negative effects, rewarding feelings such as self-esteem, self-affirmation, and life satisfaction were also reported (5), indicating the positive psychological wellbeing status that may be induced during the caregiving process. However, a recently published systematic review found that most studies focused on the negative aspects of caregiving, and positive wellbeing aspects, especially psychological wellbeing, are overlooked by research (7). As in recent years, the pool of informal caregivers has declined, and a more balanced portrayal of the health effects of caregiving is needed to encourage more people to involve in caregiving (8). Hence, more research on caregiver psychological wellbeing is needed.

Psychological wellbeing, defined as the inter- and intra individual levels of positive functioning from six constructs (i.e., self-acceptance, autonomy, personal growth, purpose in life, environmental mastery, and positive relations with others), is an important construct of caregiver health, and a facilitator for reducing mortality (9, 10). Research found that dementia caregivers' psychological wellbeing changes over time, and it gets worse with the increase of patient neuropsychiatric symptoms and caregiver burden (11). Hence, studying dementia caregivers' psychological wellbeing from the early to moderate stage of dementia is essential for maintaining wellbeing and preventing psychological problems. However, few studies have focused on this area. Very little was found in the literature on Chinese dementia caregivers' psychological wellbeing and the essential influencing factors.

Lawton's two-factor model of caregiving appraisal and psychological wellbeing indicates positive and negative paths from caregiving to wellbeing, and the essential mediator between them is caregiving appraisal (12). Caregiving appraisal is an individual's cognitive evaluation of the potential stressors caused by caregiving, as well as the evaluation of their resources and coping efforts (13). It has three forms and five constructs: positive (i.e., caregiving satisfaction and caregiving mastery), neutral (i.e., caregiving ideology), and negative appraisal (i.e., subjective caregiving burden and impact of caregiving) (13). Our systematic review found that caregiving appraisal is influenced by a cluster of factors, namely, caregivers' perceptions of the illness, coping strategies, social pressure, concerns in caregiving, etc. (14). We also identified that a significant underlying factor that may determine the influence of these factors is dementia literacy.

Dementia literacy refers to the knowledge of dementia and the attitudes toward dementia (15). In recent years, research about dementia literacy has been conducted among the general population (15) and older adults (16). However, the dementia literacy of the essential stakeholder in caregiving, i.e., dementia caregivers, is relatively unexplored. A recent publication showed that caregivers have a reasonable knowledge of dementia and a less positive attitude toward dementia (17). However, much less is known about Chinese caregivers' dementia literacy. Although extensive research has been carried out based on Lawton's model, no single study exists that measured the influence of dementia literacy on caregiving appraisal. If the mechanism behind this influence can be identified, effective strategies may be explored to improve the caregivers' psychological wellbeing.

A systematic review of qualitative studies identified that caregivers' appraisal and psychosocial outcomes are partially determined by their knowledge of dementia and their attitude toward dementia (18). A recent quantitative study also preliminarily confirmed the positive correlations between dementia knowledge and attitude, as well as the significant association among dementia knowledge, attitude toward dementia and certain constructs of caregiving appraisal (19). The positive and negative aspects of caregiving appraisal was also found to be significantly associated caregiver psychological wellbeing (20). Hence, we assume that caregivers' dementia literacy, caregiving appraisal, and psychological wellbeing correlate with each other. However, the mechanism that underpins this association is not fully understood.

So far, no research has been conducted to demonstrate the association mechanism among dementia literacy, caregiving appraisal, and psychological wellbeing. However, it can be inferred that the influence of dementia literacy on psychological wellbeing is partially mediated by caregiving appraisal by empirical evidence. Recent research found that dementia caregivers' health literacy is associated with their caregiving appraisal, especially the appraised caregiving burden (21). Lack of knowledge may hinder caregivers' adaptations to caregiving, which in turn may influence caregiver wellbeing (22). As an essential factor during the adaptation process, caregiving appraisal mediates the relationships between caregiver factors and the adaptation outcomes (23). As psychological wellbeing status is a result of adaptation, we assume that caregiving appraisal may also mediate the association between caregivers' dementia literacy and psychological wellbeing. A cross-sectional study was hence designed to test this assumption.

## Objectives

To investigate the status of the psychological wellbeing of informal caregivers of people with dementia, and the association between dementia literacy (i.e., knowledge of dementia and attitude toward dementia), caregiving appraisal, and psychological wellbeing, as well as the influencing mechanism among them.

## Hypotheses

Hypothesis 1: Dementia literacy (including dementia knowledge and attitudes toward dementia), caregiving appraisal, and psychological wellbeing are correlated.

**Figure 1:**
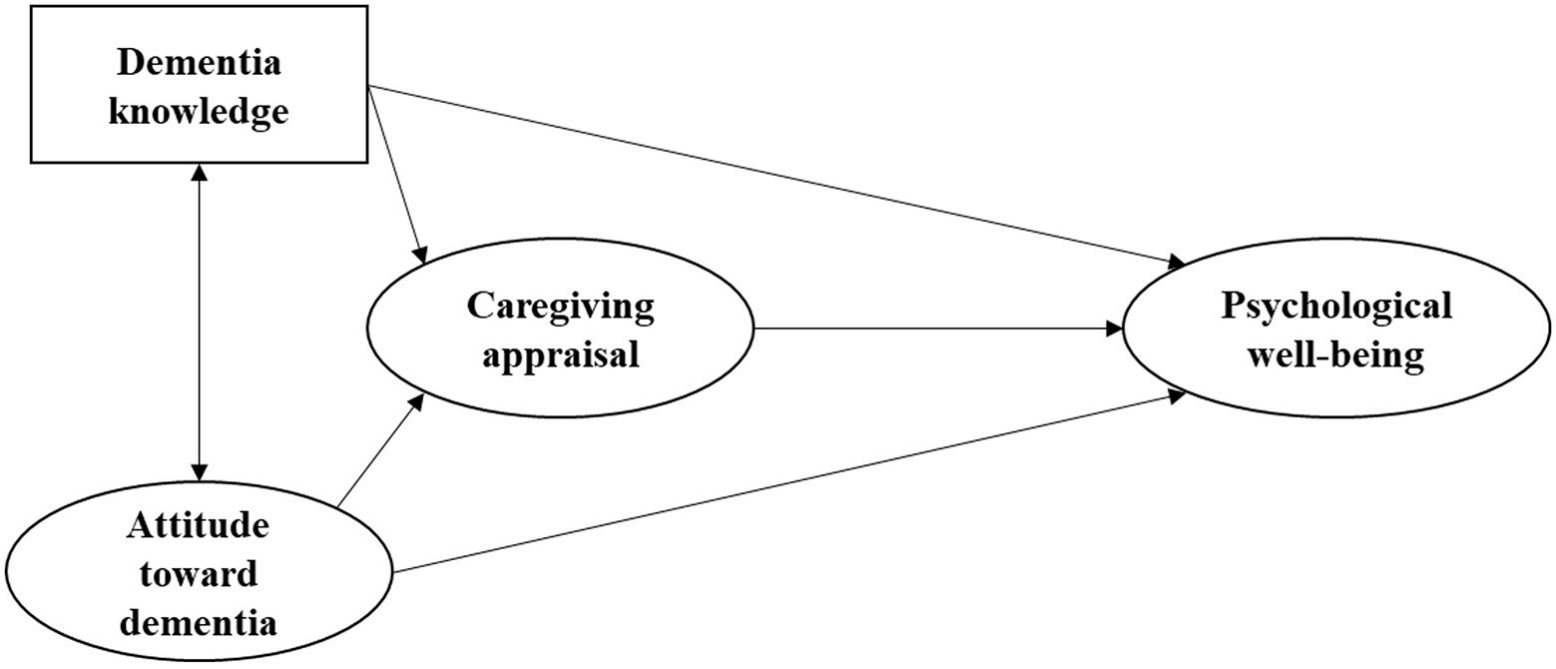
The hypothesized model based on theory and literature.

Hypothesis 2: Caregiving appraisal mediates the association between dementia literacy and psychological wellbeing (Figure 1).

The hypothesized model (Figure 1) is derived from Lawton's model (12) and evidence from empirical studies. The model assumes that the two constructs of dementia literacy (i.e., knowledge of dementia and attitude toward dementia) are correlated. They could directly influence psychological wellbeing, and indirectly influence it *via* the mediation of caregiving appraisal.

## Methods

### Design and setting

This is a cross-sectional study conducted from October 2020 to December 2021. Data were collected from the outpatient department of two teaching hospitals in Zhengzhou, China.

### Participants

Convenience sampling was used to recruit participants from the outpatient departments of the two hospitals. The inclusion criteria included: the primary caregiver who's faced with the main duty to provide unpaid care to a person with diagnosed mild to moderate dementia (Global Deterioration Scale score = 4~6); aged 18 years or older; free from any diagnosed psychiatry co-morbidity; assist with at least one of the care recipient's activities of daily living; provide care to the person with dementia for at least 6 months. The exclusion criteria included: caregivers who cannot read; caregivers who cannot answer questions logically.

The sample size calculation was based on the observation-per-parameter ratio of the hypothesis model. In the hypothesis model, 13 free parameters were estimated. Adopting the recommendation of 1–20 times free parameters (24), 15 times was chosen for this study based on the feasibility of recruitment during the pandemic. Hence, the minimum sample size should be 195. Considering an attrition rate of 20%, at least 234 eligible participants should be recruited.

### Variables and measurement

#### Dementia literacy

As there is no validated dementia literacy scale available, dementia literacy was measured with Alzheimer's Disease Knowledge Scale (25) and Dementia Attitude Scale (26), which were commonly used by previous publications (15). The Alzheimer's Disease Knowledge Scale was a 30-item *true/false* question related to dementia assessment, diagnosis, prevalence, prevention, risk factors, life impact, symptoms, treatment, caregiving, and management. A composite score could be calculated by counting the correct answers, and the total score range from 0 to 30. The Cronbach's α of this scale was 0.758. The Dementia Attitude Scale was a 20-item Likert 7-point scale. This scale includes two subscales: dementia knowledge and social comfort. Higher scores indicate a more positive attitude toward dementia. The total scale Cronbach's α = 0.754, Cronbach's α for the subscales ranged from.574 to.800.

#### Caregiving appraisal

The Caregiving Appraisal Scale was used (27). It is a 26-item Likert 5-point scale. The Chinese version scale includes two constructs and four subscales: positive caregiving appraisal (including the caregiving mastery and caregiving satisfaction subscales) and negative appraisal (caregiving burden, caregiving impact subscales). The scores of caregiving burden and caregiving impact were recoded when calculating the total scale score, so that higher scores indicate more positive caregiving appraisal. The total scale Cronbach's α = 0.801, and the Cronbach's α for the subscales ranged from 0.679 to 0.839.

#### Psychological wellbeing

The shorter version of Ryff's Psychological wellbeing Scale was used (28). This scale is an 18-item Likert 6-point scale divided into six subscales: autonomy, positive relations with others, personal growth, environmental mastery, self-acceptance, and purpose in life. Higher scores indicate better psychological wellbeing status. The total scale Cronbach's α = 0.921, and the Cronbach's α for the subscales ranged from 0.692 to 0.868.

### Data collection process

The health professionals in the hospitals, i.e., the nurse and doctor-in-charge of the department, referred patients to the research team, and the team contacted the participants during their outpatient visits. Only one primary informal caregiver of each patient was enrolled in this study. All participants were informed of the purpose and procedure of this study, and informed consent was obtained from all of them. The consented participants were interviewed by the research personnel either in person, *via* telephone, or by self-administering online questionnaire. Detailed instructions on how to fill out the questionnaire were introduced to them before conducting the survey.

### Statistical methods

R software 4.0.5 was used to do the data analysis. Descriptive statistics were used to analyze the proportion of missing values. As the proportion was lower than 5% (0.4%~1.3%), the series means were used to compute the missing values as suggested by Hair et al. (29). Descriptive statistics were used to analyze the demographics and the outcome measures. Pearson's correlation analysis was used to analyze the associate factors of psychological wellbeing. The Lavaan package for R software was used for structural equation modeling. In the structural equation modeling, attitude toward dementia, caregiving appraisal, and psychological wellbeing was used as the latent variable, and the subscales of the latent variables and total score of knowledge of dementia were used as the observed variables. Maximum likelihood estimation and absolute and relative indices were used to evaluate the goodness-of-fit indices. The values of *p*(χ^2^) >0.05, the comparative fit index (CFI) >0.90, the root mean square error of approximation (RMSEA) <0.05, goodness-of-fit index (GFI) >0.90, the adjusted goodness-of-fit index (AGFI) >0.90, normed fit index (NFI) >0.90, relative fit index (RFI) >0.90, the Tucker Lewis index (TLI) >0.90 indicate a good model fit.

### Ethical consideration

Ethical approval was obtained from the first author's university. The investigation was anonymous, and all the information collected was kept confidential. Only the research team could access the data. Informed consent was achieved from all participants.

## Results

### Demographics and descriptions of the study variables

The research team approached 352 caregivers over 15 months' period, during which 240 participants (68.18%) met our eligibility criteria and consented to participate in this study. Two hundred and forty questionnaires were distributed, and 223 (92.92%) valid questionnaires were returned and used in the analysis. No significant difference was detected between participants who completed the survey and those who did not, in terms of age, gender, education, marital status, and relationship with the care recipient.

The age of the caregivers was 57.73 (SD, 14.51). Eighty-three (37.2%) of the caregivers were male, and 140 (62.8%) of them were female. The average years of caregiving were 4.04 (SD, 3.12). The education level included illiterate (*n* = 25, 11.5%), primary education (*n* = 61, 28.1%), secondary education (*n* = 78, 35.9%), tertiary education (*n* = 53, 24.4%). 34.5% of the caregivers were working caregivers, 19.7% were unemployed, and 45.7% were retired. The relationship with the care recipient were spouse (*n* = 78, 35%), children or spouse of children (*n* = 129, 57.9%), other relatives (*n* = 16, 7.1%). The marital status of caregivers was married (*n* = 204, 93.2%) or other (*n* = 15, 6.8%).

The average age of care recipients was 76.97 (SD, 13.39). The type of dementia included Alzheimer's disease (*n* = 128, 57.4%), vascular dementia (*n* = 78, 35.0%), and other types of dementia (*n* = 17, 7.6%). One hundred three (46.2%) of the patients were male, and 120 (53.8%) were female. The stage of dementia was mild (19.7%), moderate (49.8%), and moderately severe (30.5%).

In general, the scores of dementia literacy, caregiving appraisal, and psychological wellbeing were not high. Specifically, the average correct answer fill rate of knowledge of dementia was only 61.23%, and the correct response rate of four out of seven (4/7) aspects of knowledge was lower than 60%. The average score of attitudes toward dementia, caregiving appraisal, and psychological wellbeing was 64.5, 64.7, and 74.2% of the highest possible score, respectively (Table 1).

**Table 1:** Means and standard deviation of the study variables (*n* = 223).

**Variable**	**Score range**	**Mean**	**SD**	**Correct response rate**
**Dementia literacy**
- **Knowledge of dementia**	0–30	18.37	3.35	0.61
Life impact	0–3	2.07	1.00	0.69
Treatment and management	0–4	2.72	1.23	0.68
Course	0–4	2.64	1.14	0.66
Assessment and diagnosis	0–4	2.34	1.05	0.58
Symptoms	0–4	2.25	1.14	0.56
Risk factor	0–6	3.22	1.45	0.54
Caregiving	0–5	1.83	1.02	0.37
- **Attitude toward dementia**	20–140	90.36	14.28	
Social comfort	10–70	40.47	8.48	
Dementia knowledge	10–70	49.89	8.58	
**Caregiving appraisal**	26–130	84.13	11.28	
Caregiving burden	12–60	37.75	7.34	
Caregiving impact	5–25	13.38	3.55	
Caregiving mastery	4–20	13.98	2.18	
Caregiving satisfaction	5–25	19.13	2.45	
**Psychological wellbeing**	18–108	80.11	13.70	
Positive relations with others	3–18	13.69	2.89	
Autonomy	3–18	12.47	2.91	
Environmental mastery	3–18	14.35	2.93	
Personal growth	3–18	13.36	2.98	
Purpose in life	3–18	12.62	3.16	
Self-acceptance	3–18	13.49	2.89	

### Correlation between the variables

The knowledge of dementia, attitude toward dementia, and psychological wellbeing were positively associated with each other (*p* < 0.05 or *p* < 0.01). However, knowledge of dementia was not significantly associated with caregiving appraisal (*p* > 0.05). Caregiving appraisal, attitude toward dementia, and psychological wellbeing were positively associated with each other (*p* < 0.01; Table 2). The correlations between subscales are in Supplementary Table 1. Knowledge of dementia was significantly associated with the two subscales of attitude toward dementia (*p* < 0.01), and the positive relations with others (*p* < 0.01) and self-acceptance (*p* < 0.05) subscales of psychological wellbeing. The social comfort subscale was significantly associated with all the subscales of caregiving appraisal (*p* < 0.05 or *p* < 0.01), and the environmental mastery (*p* < 0.01) and self-acceptance (*p* < 0.01) subscales of psychological wellbeing. Whereas, the dementia knowledge subscale was significantly associated with the caregiving impact (*p* < 0.01) and caregiving satisfaction (*p* < 0.01) subscales of caregiving appraisal, and all the subscales of psychological wellbeing (*p* < 0.01). In general, the correlations were weak to moderate (|*r*| = 0.135 to 0.673).

**Table 2:** Correlations between the variable total scores (*n* = 223).

**Variable**	**Knowledge of dementia**	**Attitude toward dementia**	**Caregiving appraisal**	**Psychological wellbeing**
Knowledge of dementia	1			
Attitude toward dementia	0.327**	1		
Caregiving appraisal	0.016	0.312^**^	1	
Psychological wellbeing	0.136^*^	0.311^**^	0.267^**^	1

** p <0.01.

* p <0.05 (two-tailed test).

### The mediation effects

**Figure 2:**
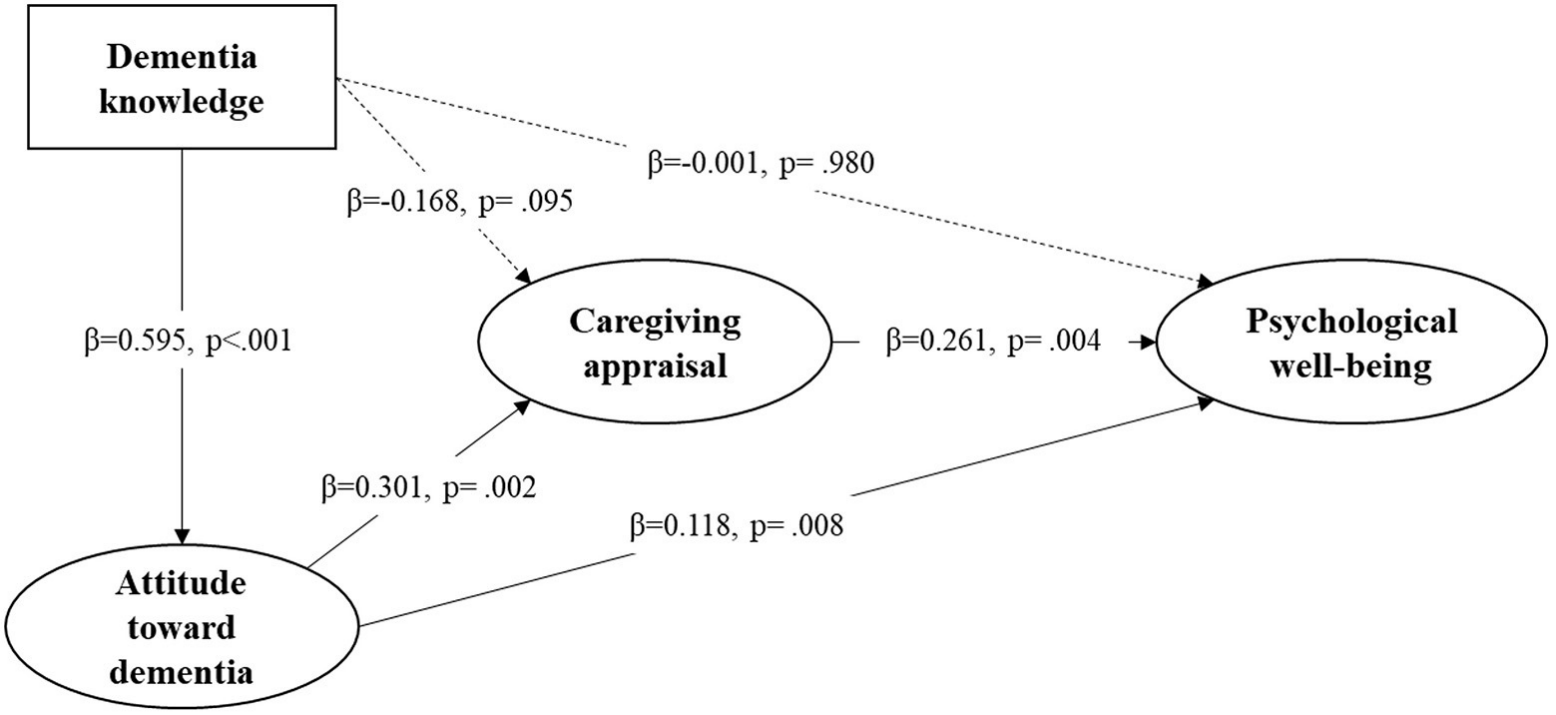
The fitted structural equation model with the influencing mechanism of study variables. χ^2^ = 44, *p* (χ^2^) = 0.078, CFI = 0.987, RMSEA = 0.038, GFI = 0.959, AGFI = 0.916, NFI = 0.949, RFI = 0.909, TLI = 0.976.

The results of structural equation modeling showed that the model generally fit well (*p* > 0.05), and all the model fit indexes met the criteria for a good model fit (Figure 2). Table 3 displays the modified model's direct, indirect, and total effects. The total effect of knowledge of dementia on psychological wellbeing was significant (β = 0.117, *p* < 0.001). Likewise, the indirect effect of knowledge of dementia on psychological wellbeing *via* attitude toward dementia and caregiving appraisal (ADKS-DAS-CA-PWB) was significant (β = 0.047, *p* = 0.018), and the indirect effect of knowledge of dementia *via* attitude toward dementia (ADKS-DAS-PWB) were also significant (β = 0.070, *p* = 0.013). However, the direct effect of knowledge of dementia on psychological wellbeing was insignificant (β = −0.001, *p* = 0.980), indicating that the influence of knowledge of dementia was fully mediated by attitude toward dementia and caregiving appraisal.

**Table 3:** Path coefficients of the modified model.

**Route**	**Estimate**	**SE**	***z***	***p*-Value**	**95%CI**	
					**Lower**	**Upper**
**Direct effect**
DAS-PWB	0.118	0.045	2.639	0.008	0.030	0.205
**Indirect effect**
ADKS-DAS-CA-PWB	0.047	0.020	2.376	0.018	0.008	0.086
ADKS-DAS-PWB	0.070	0.028	2.492	0.013	0.015	0.125
DAS-CA-PWB	0.079	0.032	2.483	0.013	0.017	0.141
**Total effect**
ADKS-PWB	0.117	0.029	4.000	<0.001	0.060	0.174
DAS-PWB	0.196	0.042	4.660	<0.001	0.114	0.279

SE, standard error; ADKS, Alzheimer's disease knowledge scale; DAS, dementia attitude scale; CA, caregiving appraisal scale; PWB, psychological wellbeing scale.

The total effect of attitude toward dementia on psychological wellbeing was significant (β = 0.196, *p* < 0.001). Likewise, the indirect effect of attitude toward dementia *via* caregiving appraisal (DAS-CA-PWB) was also significant (β = 0.079, *p* = 0.013). Considering the significant direct effect of attitude toward dementia on psychological wellbeing (β = 0.118, *p* = 0.008), we conclude that caregiving appraisal partially mediates the association between attitude toward dementia and psychological wellbeing.

## Discussion

This is one of the first studies to investigate the association between dementia literacy, caregiving appraisal, and psychological wellbeing. It is evident that caregivers in this study have limited knowledge of dementia and attitude toward dementia, similar to studies conducted in other countries (30). The psychological wellbeing status is also not very good, signifying a necessity to explore the essential influencing factors and supporting strategies to improve this situation.

Partially confirming the first hypothesis, the significant influence of dementia literacy on caregiving appraisal and psychological wellbeing was only demonstrated in attitude toward dementia. In this study, knowledge of dementia was positively associated with attitude toward dementia and psychological wellbeing, and it was identified to be a direct predictor of attitude toward dementia. Even though no caregiver evidence has been identified from previous research, this finding can be partially explained by a study conducted among physiotherapists, which indicates that the more they were educated about dementia knowledge, the more positive attitudes they would have (31). However, a different opinion was identified by a study in Ireland, which found that greater knowledge of dementia does not ensure a positive attitude toward dementia among the general public (32). The inconsistent findings arouse more research to make a firm conclusion in this regard.

In testing the second hypothesis, an interesting finding was found that even though caregivers do not have enough knowledge of dementia, the knowledge per se did not directly influence their caregiving appraisal and psychological wellbeing. Knowledge could only influence appraisal and psychological wellbeing *via* the mediation of attitude toward dementia. This finding indicates that even when caregivers have limited knowledge of the illness, they can still have a sense of positive appraisal, such as satisfaction and mastery of the caregiving tasks. It may be because dementia is a long-term condition with no cure. Caregivers' appraisals are more influenced by the stressors or supporting factors that could possibly be modified (such as patient behavioral problems, social support, or coping skills) rather than the illness characteristics that are not truly related to their caregiving (such as the assessment, diagnosis, course, and risk factors of dementia) (33). It also indicates that, in this association, attitude plays a more significant role than knowledge. Possible reasons may be the unique social feature of dementia. Globally, dementia social stigma is very common. Specifically, people who have limited knowledge of dementia tend to show stigmatizing attitudes toward dementia (34). Some people even treat having dementia as being crazy, witches, or the will of God, which may influence their appraisal and psychological wellbeing (35).

The essential mediating role of caregiving appraisal was demonstrated, but the mediation pathway differed from the hypothesis. Caregiving appraisal and attitude toward dementia fully mediated the association between knowledge of dementia and psychological wellbeing. It also partially mediated the association between attitude toward dementia and psychological wellbeing. This finding reflected that both aspects of dementia literacy could influence psychological wellbeing through changing caregiving appraisal, especially for knowledge of dementia, which could only influence psychological wellbeing *via* the mediation effect. Although no previous research on dementia was found, the finding was in line with the findings of a systematic review, which indicated that caregivers' inadequate health literacy was associated with poor health-related behaviors and outcomes (36).

Even though some innovative findings have been identified, several limitations of this study still need to be considered, indicating implications for future research. Random sampling was less feasible during the pandemic, so convenient sampling was used to recruit participants. Selection bias cannot be avoided, inducing limited representativeness. Future studies could consider random sampling and bigger sample size to increase the perceptiveness and generalizability. Second, self-reported questionnaires were used to collect data. Even though the research team provided detailed guidance on how to fill out the questionnaires, socially desirable answers cannot be eliminated. Third, Alzheimer's disease knowledge scale and attitude toward dementia scale were used to measure dementia literacy because there is a lack of validated instruments designed specifically for Chinese caregivers' dementia literacy. Potential constructs of dementia literacy, such as specific dementia services, were not measured in this study. Future studies can develop a comprehensive, validated instrument.

This study also has some implications for practice. Dementia caregivers showed limited dementia literacy, which signifies the necessity for more dementia-related education for caregivers. Healthcare professionals are suggested to incorporate information about dementia literacy in the health education to caregivers. As caregivers' attitudes toward dementia may be influenced by social stigma, social media can be used to increase public awareness of dementia, decrease social stigma toward dementia, and improve the public's attitude toward dementia. In light of the significant mediation effect of caregiving appraisal, future research and practice are also suggested to design strategies to improve caregiving appraisal, so that the effect of psychosocial education on improving psychological wellbeing can be boosted.

## Conclusion

This study contributed new knowledge to dementia research by analyzing the influencing mechanism among dementia literacy, caregiving appraisal, and psychological wellbeing. The findings demonstrated that informal caregivers of people with dementia have unsatisfactory dementia literacy and psychological wellbeing. Attitude toward dementia, caregiving appraisal, and psychological wellbeing are significantly correlated. However, knowledge of dementia was not significantly associated with caregiving appraisal. Attitude toward dementia could directly influence psychological wellbeing, or *via* the mediation of caregiving appraisal. Knowledge of dementia could only influence psychological wellbeing *via* the mediation of attitude toward dementia and caregiving appraisal. This study suggests that improving attitude toward dementia and caregiving appraisal could help improve the psychological wellbeing of informal caregivers of people with dementia.

## Data availability statement

The data is published in the Supplementary file. Further inquires can be directed to the corresponding author.

## Ethics statement

The studies involving human participants were reviewed and approved by School of Nursing and Health, Zhengzhou University. The patients/participants provided their written informed consent to participate in this study.

## Author contributions

SW was responsible for the research design and drafting. QS contributed to the study implementation. IL was responsible for the statistical support and data analysis. DC, XX, and AL gave constructive comments on this manuscript. All the authors reviewed the manuscript and approved the submission.

## Conflict of interest

The authors declare that the research was conducted in the absence of any commercial or financial relationships that could be construed as a potential conflict of interest. The reviewer AC declared a shared affiliation with the authors SW, DC, and AL to the handling editor at the time if the review.

## Publisher's note

All claims expressed in this article are solely those of the authors and do not necessarily represent those of their affiliated organizations, or those of the publisher, the editors and the reviewers. Any product that may be evaluated in this article, or claim that may be made by its manufacturer, is not guaranteed or endorsed by the publisher.
