# Supplementary material for: Influence of dementia literacy and caregiving appraisal on the psychological wellbeing of informal caregivers of people with dementia: A cross-sectional study
Source: Front Med (Lausanne). 2022 Sep 14;9:971481. doi: 10.3389/fmed.2022.971481 (PMC9515462; doi:10.3389/fmed.2022.971481)
Supplement: Supplementary file 1 [file Table_1.DOCX]

**Supplementary Table 1** Correlations between the subscales (n=223)

|  | 1 | 2 | 3 | 4 | 5 | 6 | 7 | 8 | 9 | 10 | 11 | 12 | 13 |
| --- | --- | --- | --- | --- | --- | --- | --- | --- | --- | --- | --- | --- | --- |
| 1. Knowledge of dementia | 1 |  |  |  |  |  |  |  |  |  |  |  |  |
| 2. Social comfort | .239^**^ | 1 |  |  |  |  |  |  |  |  |  |  |  |
| 3. Dementia knowledge | .308^**^ | .403^**^ | 1 |  |  |  |  |  |  |  |  |  |  |
| 4. Caregiving burden | .017 | -.267^**^ | -.093 | 1 |  |  |  |  |  |  |  |  |  |
| 5. Caregiving impact | -.015 | -.286^**^ | -.174^**^ | .651^**^ | 1 |  |  |  |  |  |  |  |  |
| 6. Caregiving mastery | .111 | .155^*^ | .055 | .103 | -.038 | 1 |  |  |  |  |  |  |  |
| 7. Caregiving satisfaction | .072 | .293^**^ | .230^**^ | -.304^**^ | -.308^**^ | .497^**^ | 1 |  |  |  |  |  |  |
| 8. Positive relations with others | .266^**^ | .091 | .355^**^ | -.009 | -.130 | .243^**^ | .319^**^ | 1 |  |  |  |  |  |
| 9. Autonomy | .090 | .129 | .268^**^ | .056 | -.104 | .255^**^ | .135^*^ | .376^**^ | 1 |  |  |  |  |
| 10. Environmental mastery | .118 | .233^**^ | .357^**^ | -.176^**^ | -.290^**^ | .381^**^ | .368^**^ | .482^**^ | .552^**^ | 1 |  |  |  |
| 11. Personal growth | .064 | .083 | .272^**^ | -.181^**^ | -.191^**^ | .179^**^ | .267^**^ | .503^**^ | .452^**^ | .673^**^ | 1 |  |  |
| 12. Purpose in life | -.031 | -.001 | .213^**^ | -.102 | -.024 | .144^*^ | .247^**^ | .385^**^ | .368^**^ | .555^**^ | .646^**^ | 1 |  |
| 13. Self-acceptance | .155^*^ | .225^**^ | .284^**^ | -.056 | -.173^**^ | .315^**^ | .296^**^ | .521^**^ | .515^**^ | .664^**^ | .584^**^ | .514^**^ | 1 |

*Notes:* **. Correlation is significant at the 0.01 level (2-tailed), *. Correlation is significant at the 0.05 level (2-tailed).
